# 

**Published:** 1883-08-20

**Authors:** 


### TABLE OF CONTENTS.

ORIGINAL AND SELECTED ARTICLES.

Hemorrhagic Malarial Fever, by E. H. M. Parham, M.D., of Arkansas, 281. Uremia, by J. H. Low, M.D., of New York, 284. New York Medical and Surgical Society, 285. Cholera—What is it? by Prof. T. S. Bell, M.D., 289. Pharmaceutical Preparations of Corn Silk, by George W. Kennedy, Ph. D., 294. Iodide of Potassium, by George Thomas Jackson, M. D., 296.

ABSTRACTS AND GLEANINGS.

Practical Medicine, 302. Deaths During the Administration of Anæsthetics, 303. Successful Laparotomy for Intestinal Obstruction, 305. Extraction of Deciduous Teeth, 306. Calomel in Croup—Colchicum in Gout, 307. Don't, 308. Treatment of Nasal Catarrh; Case of Stramonium Poisoning, 309. Kairine, a Substitute for Quinine; A Dentist's Fee, 310.

SCIENTIFIC ITEMS.

The Gradual Cooling of the Earth; The Effect of the Castor Oil Plant on Flies; Octagonal Religious Services in the Eastern Penitentiary, Philadelphia; The Coldest Town in the World, 311. Tremors of the Earth; Vaccinating Live Stock; An Expert Machinest; Large Telephone, 312.

PRACTICAL NOTES AND FORMULÆ.

Compound Syrup of Wild Cherry; Blackberry Cordial; New Use of Chloral, 313. Hop Cordial; Brown's Troches; Laxative in Childbed; Flatulent Dyspepsia, 314. For Pain Preceding the Menstrual Flow; Oxide of Zinc in Chronic Diarrhœa; Liniments in Muscular Rheumatism; Dysmenorrhœa Mixture, 315.

EDITORIALS AND MISCELLANEOUS.

Editorial Notices; Practical Anatomy, 316. Medical Ministry, 317. St. Louis Medical Society; Transactions of the Medical Society of Tennessee; Transactions of the Mississippi State Medical Association, 318. Transactions of the Medical Society of the State of West Va.; Pamphlets Received, 319. Receipted; Special Notices, 320.

